# Balloon aortic valvuloplasty (BAV) as a bridge to aortic valve replacement in cancer patients who require urgent non-cardiac surgery

**DOI:** 10.2478/raon-2013-0078

**Published:** 2014-01-22

**Authors:** Polonca Kogoj, Rok Devjak, Matjaz Bunc

**Affiliations:** 1 Department of Cardiology, Division of Internal Medicine, University Medical Centre Ljubljana, Slovenia; 2 Institute of Oncology Ljubljana, Ljubljana, Slovenia

**Keywords:** aortic valve stenosis, balloon dilatation, angioplasty, heart valve prosthesis implantation, elderly, comorbidities, coronary artery disease

## Abstract

**Background:**

Balloon aortic valvuloplasty (BAV) is a percutaneous treatment option for severe, symptomatic aortic stenosis. Due to early restenosis and failure to improve long term survival, BAV is considered a palliative measure in patients who are not suitable for open heart surgery due to increased perioperative risk. BAV can be used also as a bridge to surgical or transcatheter aortic valve implantation (TAVI) in haemodinamically unstable patients or in patients who require urgent major non-cardiac surgery.

**Patients and methods.:**

We reported on 6 oncologic patients with severe aortic stenosis that required a major abdominal and gynaecological surgery. In 5 cases we performed BAV procedure alone; in one patient with concomitant coronary artery disease we combined BAV and percutaneous coronary intervention (PCI).

**Results:**

With angioplasty and BAV we achieved a good coronary artery flow and an increase in aortic valve area without any periprocedural complications. After the successful procedure, we observed a hemodynamic and symptomatic improvement. As a consequence the operative risk for non-cardiac surgery decreased and the surgical treatment of cancer was done without complications in all the 6 cases.

**Conclusions:**

BAV can be utilized as a part of a complex therapy in severe aortic stenosis aimed to improve the quality of life, decrease the surgical risk for major non-cardiac surgery or as a bridge to surgical or transcatheter aortic valve implantation.

## Introduction

### Aortic stenosis in high risk patients

Calcific stenosis of the aortic valve (AS) is the most common acquired valve disorder in the Western world.^1^ It is a degenerative, atherosclerosis-like chronic inflammatory process that leads to lipid and calcium accumulation into the valve leaflets. It leads to leaflet sticking, limitation of movement and narrowing of the aortic valve area (AVA). The disease progresses slowly and the prevalence is increasing with aging of the population. Moderate AS is present in 2 to 7% of the population over 65 years and severe AS is present in 5 to 7% of the population over 80 years and in 10 to 15% over 90 years.^1^–^3^ Patients may be asymptomatic for several years and become symptomatic only in the last stage of the disease when the average survival is around 2 years with high risk for sudden cardiac death.^4^ So far no reliable data exist on prevalence of AS in patients with cancer but this coincidence is not rare in clinical practice.

Surgical aortic valve replacement (SAVR) is considered the treatment of choice in patients with severe, symptomatic AS regardless of age.^5^,^6^ The surgical risk in elderly patients with multiple co-morbidities can be very high and the presence of concomitant coronary artery disease (CAD) with the need for additional coronary artery bypass may duplicate the risk.^7^–^9^ Therefore approximately one third of such patients might not be surgically treated and are left to the natural history of the disease.^4^

### Aortic stenosis in cancer patients

The decision for SAVR is particularly complex in cancer patients where cancer prognosis and possible perioperative complications raise concerns. Thus, one of the common reasons for declining surgery in patients with severe aortic stenosis is cancer.^4^

Two studies were published on the latter, where in the first study the authors observed greater increased perioperative mortality and morbidity in chronic lymphocytic leukaemia patients after an open heart surgery, due to infectious complications.^10^ The second study did not prove this kind of phenomena, but, the authors included patients with solid tumours, who may have less compromised immunity than patients with haematological malignancies.^11^ Yusuf *et al*. recently analyzed a group of 48 cancer patients with severe AS where 13 patients underwent SAVR and the others were managed medically. He demonstrated that cancer patients with severe AS who underwent SAVR had longer survival, regardless of cancer status or presence of metastasis.^11^

### Balloon aortic valvuloplasty

**FIGURE 1.**
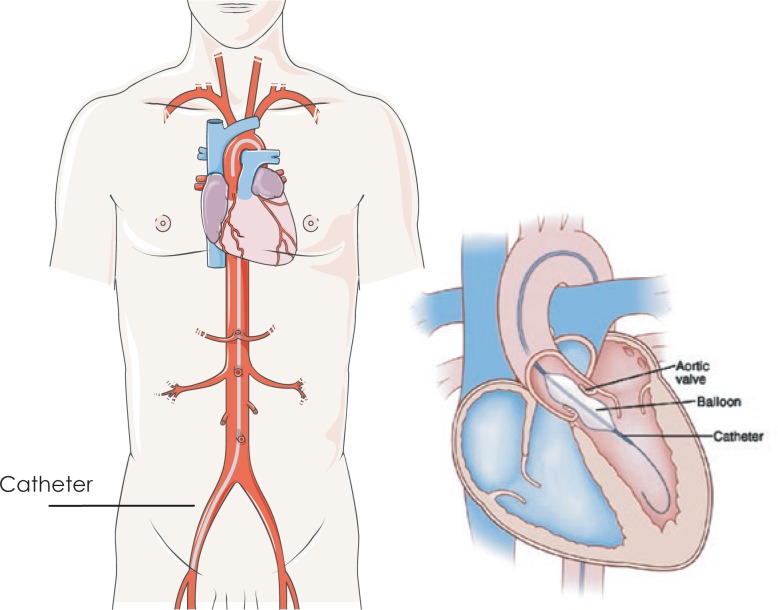
Balloon aortic valvuloplasty (BAV): The balloon catheter is advanced via femoral artery to the left ventricle and placed into the stenotic aortic valve where the balloon is inflated.

Balloon aortic valvuloplasty (BAV) was introduced in 1986 as the first less invasive, percutaneous treatment option for treatment of AS (Figure 1).^12^

Unfortunately, early restenosis of the dilated valve with symptom recurrence and poor long term survival limits the use of this procedure.^13^–^17^ Today BAV is considered a palliative measure in patients with increased perioperative risk or a bridge to open heart surgery in haemodinamically unstable patients or in patients who require urgent major non-cardiac surgery, like oncologic patients. In case of concomitant coronary artery disease (CAD), valvuloplasty and coronary angioplasty (PCI) may be performed safely during the same procedure.^18^,^19^ In the last few years new therapeutic options are being developed such as transcatheter aortic valve implantation (TAVI) where BAV plays an important role in preparing the stenotic aortic valve for the aortic valve prosthesis implantation.^20^–^23^ Furthermore, in high risk patients where SAVR is not an option BAV can be used as a bridge to TAVI.

In this paper we report experiences of a single institution in percutaneous treatment of aortic stenosis in oncologic patients undergoing urgent non-cardiac surgery.

## Patients and methods

We retrospectively reviewed all the patients who were treated with BAV in the Department of Cardiology, Division of Internal Medicine, University Medical Centre Ljubljana from June 2009 to April 2011. We included in the present study only the high risk patients who presented with carcinoma and required the procedure before urgent major tumour excision.

Inclusion criteria for BAV were:

- severe AS,
- increased perioperative risk defined with Logistic EuroSCORE (European System for Cardiac Operative Risk Evaluation calculated at http://www.euroscore.org/) > 20%,
- indication for urgent major tumour excision

### Description of the procedure

Before and after BAV patients underwent invasive and non invasive cardiac diagnostic studies to evaluate the severity of aortic stenosis and left ventricular function. Informed consent was obtained from all the involved patients and their relatives. The procedure was performed in our cardiac catheterisation laboratory in local anaesthesia. Via percutaneous transfemoral approach a balloon catheter (18–23 mm × 4.0 cm) was introduced and positioned across the stenotic aortic valve (Figure 1). Aortic valvuloplasty was performed with balloon inflation (25–30 ml, 3–4 atm) with the aim to increase AVA and reduce transaortic pressure gradient. Before and after the valvuloplasty the peak to peak pressure gradient was measured with the pigtail catheter. The goal of the procedure was a reduction of the pressure gradient by at least 50% and if necessary, the balloon inflation could be repeated. Before valvuloplasty a coronary angiography was performed and in case of CAD, angioplasty was also done during the same procedure.

## Results

Among 230 patients who underwent BAV since June 2009, we performed the procedure in 6 high risk patients who presented with severe aortic stenosis and required an urgent non-cardiac surgery. Baseline characteristics and the procedure results are displayed in Table 1. The patients were at high risk for surgery with mean age of 79.7 years and mean logistic EuroSCORE of 12.37%. Five of them suffered from gastrointestinal and one from gynaecological carcinoma. In one patient we observed concomitant obstructive CAD that was resolved with PCI and implantation of two coronary stents, in the rest of the patients only BAV was done.

Echocardiographic assessment before and after BAV showed a significant increase in AVA (from 0.67 to 0.78 cm^2^; p<0.05) accompanied by a decrease in peak and mean transvalvular pressure gradients (from 68 to 54 mmHg; p<0.05 and from 48 to 39 mmHg; p<0.05 respectively). No significant change in left ventricular systolic function evaluated with left ventricular ejection fraction (EF) was noted (EF from 56.0% to 58.8%; p = 0.16). We did not observe any periprocedural death and any severe periprocedural complication. In one case the balloon catheter got stuck in left femoral artery and was removed surgically. In another patient with a known coronary artery disease, we observed a new onset of left bundle branch block with troponin elevation. In this patient we repeated coronarography and we excluded new lesions on coronary arteries. The average duration of hospitalization was 5.7 days. Soon after the percutaneous procedure the patients underwent surgery: 4 patients gastrointestinal tract resection, one abdominoperineal re-section and right adnexectomy and one vulvar re-section. The surgery was successful in all six cases without cardiovascular complications.

The median follow up period was 18.5 months. Successful procedure was associated with early symptomatic improvement with the decrease of New York Heart Association (NYHA) functional status from 3.2 to 2.5 and in a decrease in number of hospitalizations for cardiovascular causes. Two patients died, one 3 moths after BAV and one 10.5 months after BAV. The causes of both deaths were not related to the procedure.

## Discussion

BAV procedure was originally proposed as an alternative to SAVR for severe, symptomatic AS, but it was rapidly neglected secondary to high restenosis rates and lower survival at follow up compared to SAVR.^12^–^17^ However, BAV might be a reasonable approach to offer symptomatic relief and improvement of the quality of life in selected high risk patients when SAVR or TAVI are not an option. The new guidelines support this statement (recommendation Class IIb) and suggest this percutaneous treatment also as a bridge to SAVR or TAVI in haemodinamically unstable patients or in patients who require urgent major non-cardiac surgery.^5^,^6^

Soon after BAV we often observe an improvement of hemodynamic conditions; an increase of cardiac output, a reduction of pulmonary pressure and improvement of other heart failure clinical presentations.^24^,^25^

However, the effects of BAV are transient and usually last from three to six months. Therefore, the timescale between BAV and potentially planned non-cardiac surgery should be optimized. Patients with calcific AS often suffer also for CAD that aggravates their symptoms and contributes to the increase of surgery risk. We proved the BAV combined with PCI has no higher complication rate comparing to BAV as a single procedure.^19^

In the upper paper we describe 6 oncologic patients that underwent major abdominal and gynaecological surgery soon after BAV; in one case we combined BAV and PCI. In our cases surgery was performed without major cardiovascular complications.

Advances in cancer therapy have lead to improved survival and cancer is increasingly being recognized as a chronic disease.^26^ A recent study demonstrated that cancer patients with non-treated severe AS had worse survival in comparison to patients where the stenotic valve was surgically replaced.^11^ This suggests that AS is a condition that needs to be managed in oncologic patients as well. In selected, high risk carcinoma patients BAV may be used as bridge to TAVI and in case there is no carcinoma relapse in a year after non-cardiac surgery, may be implanted percutaneously.

## Conclusions

BAV is a feasible and safe palliative treatment for high risk patients with severe, symptomatic AS. It may be also a therapeutic bridge to SAVR or TAVI or in case of urgent non-cardiac surgery. Treatment of aortic stenosis in oncologic patients is a challenge. Percutaneous methods such as BAV should be considered as one of the treatment options in an individualized therapeutic plan that should be discussed by cardiac-oncologic team.

## Figures and Tables

**TABLE 1.** Baseline clinical characteristics and results of the procedure for each patient

**Patient number**	**1.**	**2.**	**3.**	**4.**	**5.**	**6.**
**Age (year)**	73	87	79	80	77	82
**Gender**	F	F	M	M	F	F
**Logistic EuroSCORE (%)**	5.22	12.8	5.6	28.9	5.8	15,9
**Comorbidities**	AH, HLP	AH	AH,DM,CKD,PH, ACI stenosis, HLP	PH, HF	ACI stenosis AH,HLP	CKD-HD,AH
**CAD**	NO	NO	1 VD: S/P PCI D1	NO	1 VD: M1	NO
**Carcionoma/stage**	Colon T3 N0 M0	Rectum T4 N1 M0	Colon T3 N0 MX	Rectum T3 N1 M0	Gastric T3 N0 MX	Vulva T1b N0 M0
**Suregery after BAV**	Hemicolectomy	APE and right adnexectomy	Colon resection	Abscess drainage, ileum resection	Gastric resection	Vulva resection
**Procedure**	BAV	BAV	BAV	BAV	BAV and PCI M1	BAV
**LVEF (%) before BAV**	80	60	50–55	20–25	60	60
**LVEF (%) after BAV**	85	60	55	20–25	70	60
**AVA before (cm2)**	0.9	0.8	0.67	0.45	0,7	0,5
**AVA after (cm2)**	1.0	1.1	0.7	0,55	0,75	0,6
**Peak grad before (mmHg)**	60	75	65	51	71	90
**Peak grad. after (mmHg)**	53	54	47	40	60	75
**Mean gradiet before**	42	53	40	55	53	50
**Mean gradiet after**	33	50	30	40	42	40
**Periprocedural Complications**	NO	NO	LBB, TnI	NO	NO	Catheter stuck in femoral a.
**Days of hospitalisation**	9	2	8	1	13	1
**Follow up - period months**	10.5-†	29	29	3-†	23	14

ACI = internal carotid artery; APE = abdominoperineal resection; AH = arterial hypertension; BAV = balloon aortic valvuloplasty; CAD = coronary artery disease, CKD = chronic kidney disease; DM = diabetes mellitus; D1 = first diagonal coronary artery; F = female; HD = haemodialysis; HF = heart failure; HLP = hyperlipidemia; LBB = left bundle branch block; M = male; M1 = first marginal coronary artery; PCI = percutaneous coronary intervention; PH = pulmonary hypertension; TnI: troponin I; 1 VD = one vessel disease;

† = has died during the follow up

## References

[1] Iung B, Baron G, Butchart EG, Delahaye F, Gohlke-Barwolf C, Levang OW (2003). A prospective survey of patients with valvular heart disease in Europe: The Euro Heart Survey on Valvular Heart Disease. Eur Heart J.

[2] Poredoš P (2004). Zdravstveni problemi starostnikov. Zdrav Vest.

[3] Pedersen WR, Klaassen PJ, Pedersen CW, Wilson JA, Harris KM, Goldenberg IF (2008). Comparison of outcomes in high-risk patients>70 years of age with aortic valvuloplasty and percutaneous coronary intervention versus aortic valvuloplasty alone. Am J Cardiol.

[4] Bach DS, Cimino N, Deeb GM (2007). Unoperated patients with severe aortic stenosis. J Am Coll Cardiol.

[5] Vahanian A, Alfieri O, Andreotti F, Antunes MJ, Barón-Esquivias G, Baumgartner H (2012). Guidelines on the management of valvular heart disease (version 2012): The Joint Task Force on the Management of Valvular Heart Disease of the European Society of Cardiology (ESC) and the European Association for Cardio-Thoracic Surgery (EACTS). Eur Heart J.

[6] Bonow RO, Carabello BA, Chatterjee K, de Leon AC, Faxon DP, Freed MD (2006). ACC/AHA 2006 guidelines for the management of patients with valvular heart disease: a report of the American College of Cardiology/American Heart Association Task Force on Practice Guidelines (Writing committee to revise the 1998 guidelines for the management of patients with valvular heart disease) developed in collaboration with the Society of Cardiovascular Anesthesiologists endorsed by the Society for Cardiovascular Angiography and Interventions and the Society of Thoracic Surgeons. Circulation.

[7] Goodney PP, O’Connor GT, Wennberg DE, Birkmeyer JD (2003). Do hospitals with low mortality rates in coronary artery bypass also perform well in valve replacement?. Ann Thorac Surg.

[8] Bose AK, Aitchison JD, Dark JH (2007). Aortic valve replacement in octogenarians. J Cardiothorac Surg.

[9] (2010). STS national database. STS U.S. cardiac surgery database. 1997 Aortic valve replacement patients: preoperative risk variables.

[10] Samuels LE, Kaufman MS, Morris RJ, Styler M, Brockman SK (1999). Open heart surgery in patients with chronic lymphocytic leukemia. Leuk Res.

[11] Yusuf SW, Sarfaraz A, Durand JB, Swafford J, Daher IN (2011). Management and outcomes of severe aortic stenosis in cancer patients. Am Heart J.

[12] Pai RG, Kapoor N, Bansal RC, Varadarajan P (2006). Malignant natural history of asymptomatic severe aortic stenosis: benefit of aortic valve replacement. Ann Thorac Surg.

[13] Cribier A, Savin T, Saoudi N, Rocha P, Berland J, Letac B (1986). Percutaneous transluminal valvuloplasty of acquired aortic stenosis in elderly patients: an alternative to valve replacement?. Lancet.

[14] Bashore TM, Berman AD, Davidson CJ, Mickel MC, Kennedy W, Davis KB (1991). Percutaneous balloon aortic valvuloplasty. Acute and 30-day follow-up results in 674 patients from the NHLBI Balloon Valvuloplasty Registry. Circulation.

[15] Otto CM, Mickel MC, Kennedy JW, Alderman EL, Bashore TM, Block PC (1994). Three-year outcome after balloon aortic valvuloplasty. Insights into prognosis of valvular aortic stenosis. Circulation.

[16] Davidson CJ, Harrison JK, Leithe ME, Kisslo KB, Bashore TM (1990). Failure of aortic balloon valvuloplasty to result in sustained clinical improvement in patients with depressed left ventricular function. Am J Cardiol.

[17] Shareghi S, Rasouli L, Shavelle DM, Burstein S, Matthews RV (2007). Current results of balloon aortic valvuloplasty in high-risk patients. J Invasive Cardiol.

[18] McKay RG, Safian RD, Berman AD, Diver DJ, Weinstein JS, Wyman RM (1987). Combined percutaneous aortic valvuloplasty and transluminal coronary angioplasty in adult patients with calcific aortic stenosis and coronary artery disease. Circulation.

[19] Kogoj P, Ambrožič J, Zorman D, Bunc M (2011). Percutaneous coronary intervention and balloon aortic valvuloplasty in severe aortic stenosis and coronary artery disease in elderly. [Abstract]. Int J Cardiol.

[20] Cribier A, Eltchaninoff H, Bash A, Borenstein N, Tron C, Bauer F (2002). Percutaneous transcatheter implantation of an aortic valve prosthesis for calcific aortic stenosis: first human case description. Circulation.

[21] Leon MB, Smith CR, Mack M, Miller DC, Moses JW, Svensson LG, PARTNER Trial Investigators (2010). Transcatheter aortic-valve implantation for aortic stenosis in patients who cannot undergo surgery. N Engl J Med.

[22] Smith CR, Leon MB, Mack MJ, Miller DC, Moses JW, Svensson LG, PARTNER Trial Investigators (2011). Transcatheter versus surgical aortic-valve replacement in high-risk patients. N Engl J Med.

[23] Kodali SK, Williams MR, Smith CR, Svensson LG, Webb JG, Makkar RR, PARTNER Trial Investigators (2012). Two-year outcomes after transcatheter or surgical aortic-valve replacement. N Engl J Med.

[24] Ben-Dor I, Maluenda G, Dvir D, Barbash IM, Okubagzi P, Torguson R (2013). Balloon aortic valvuloplasty for severe aortic stenosis as a bridge to transcatheter/surgical aortic valve replacement. Catheter Cardiovasc Interv 2012.

[25] Ben-Dor I, Pichard AD, Satler LF, Goldstein SA, Syed AI, Gaglia MA (2010). Complications and outcome of balloon aortic valvuloplasty in high-risk or inoperable patients. JACC Cardiovasc Interv.

[26] Velenik V, Ocvirk J, Oblak I, Anderluh F (2012). Cetuximab in preoperative treatment of rectal cancer – term outcome of the XERT trial. Radiol Oncol.

